# Nudging patients with chronic kidney disease at screening to visit physicians: A protocol of a pragmatic randomized controlled trial

**DOI:** 10.1016/j.conctc.2019.100429

**Published:** 2019-08-16

**Authors:** Shingo Fukuma, Tatsuyoshi Ikenoue, Shusaku Sasaki, Yusuke Saigusa, Toshihiro Misumi, Yoshiyuki Saito, Yukari Yamada, Rei Goto, Masataka Taguri

**Affiliations:** a Human Health Sciences, Kyoto University Graduate School of Medicine, Kyoto, Japan; b Graduate School of Economics, Kyoto University, Kyoto, Japan; c Department of Biostatistics, Yokohama City University School of Medicine, Yokohama, Japan; d Graduate School of Business Administration, Keio University, Tokyo, Japan; e Department of Data Science, Yokohama City University School of Data Science, Yokohama, Japan

**Keywords:** Nudge, Behavioral intervention, Pragmatic trial, Chronic kidney disease, Screening, Visiting behavior, CKD, chronic kidney disease, RCT, randomized controlled trial, eGFR, estimated glomerular filtration rate, ESRD, end-stage renal disease

## Abstract

**Background/Aims:**

Strategies for an effective intervention after chronic kidney disease (CKD) screening have not been well examined. We describe the rationale and design of a protocol of a pragmatic randomized controlled trial (RCT) to test the effect of a behavioral intervention using the nudge approach in behavioral economics on CKD patients’ visiting behaviors to physicians and change in their kidney function after CKD screening.

**Methods:**

The RCT will include CKD patients (N = 4500) detected at screening (estimated glomerular filtration rate [eGFR] <60 mL/min/1.74 m^2^ or urine protein ≥1+), aged 40–63 years. The two intervention groups will receive a “usual letter” and “nudge-based letter,” while the control group will only receive a conventional follow-up. Our primary outcome is proportion of patients’ visiting physicians for 6 months after the intervention; the secondary outcome is change in the eGFR at 2 years after the intervention.

**Results:**

We developed an efficient intervention program after CKD screening and designed the pragmatic RCT to assess its effectiveness in the real world. Our trial is unique in that it investigates the effect of the nudge approach in behavioral economics. By the end of 2018, we have enrolled 1,692 participants, and randomized 677 participants into the usual letter group, 677 participants into the nudge-based letter group, and 338 participants into the control group. We have confirmed that health checkup data could identify a large number of eligible participants.

**Conclusion:**

The trial's results will contribute to filling in the gap between screening and subsequent medical interventions for preventing CKD progression.

## 1 Introduction

Chronic kidney disease (CKD) is recognized as a major public health burden because it is associated with cardiovascular disease and mortality, as well as progression to end-stage renal disease (ESRD) [[1], [2], [3]]. From the perspective of health care resource utilization, a severer CKD stage is associated with higher health care cost, which is a big burden for society and the health care system [4,5]. Therefore, prevention of CKD progression is required worldwide. The first step for prevention is to identify potential CKD patients from the general population. Previous studies reported a substantial prevalence of CKD in the general population [2]; for example, 13% in Japan [6] and 11% in the United States [7].

There is a large gap between the number of potential CKD patients estimated from the laboratory results at screening and the number of diagnosed CKD patients extracted from medical records. This gap means that many of the potential CKD patients do not visit physicians to receive care for CKD. In Japan, where large-scale CKD screening is performed within the health care system [8], it is possible to clarify the actual CKD prevalence during screening of the general population and to track CKD patients' visiting behavior to physicians after CKD screening. If CKD is suspected at the health checkup based on renal function test results (urine test result and estimated glomerular filtration [eGFR]), it is recommended that patients visit a physician to confirm the presence of CKD. To maximize the effect of screening on the prevention of CKD progression, an effective intervention needs to be designed for patients suspected of having CKD in order to encourage them to appropriately visit their physicians after screening. In conventional follow-up after CKD screening in Japan, participants will be notified of their results, but there is no special intervention for offering CKD care. The intervention at that time is preferable to not be mandatory; instead, a “nudge” that respects the patient's free will is required [9]. The idea of behavioral economics and nudge approaches to healthcare is popular in public health [10].

In order to implement research to improve patients' visiting behavior to physicians after CKD screening in the general population, we launched the pragmatic trial of nudging CKD patients after screening. The trial was designed in the Japanese health care system, which has features of a universal health care system and large-scale CKD screening. The aim of the trial is to evaluate whether a behavioral intervention (i.e., a usual letter and nudge-based letter) would improve CKD patients’ visiting behaviors to physicians and protect their kidney function.

## 2 Methods

### 2.1 Overall study design

**Fig. 1:**
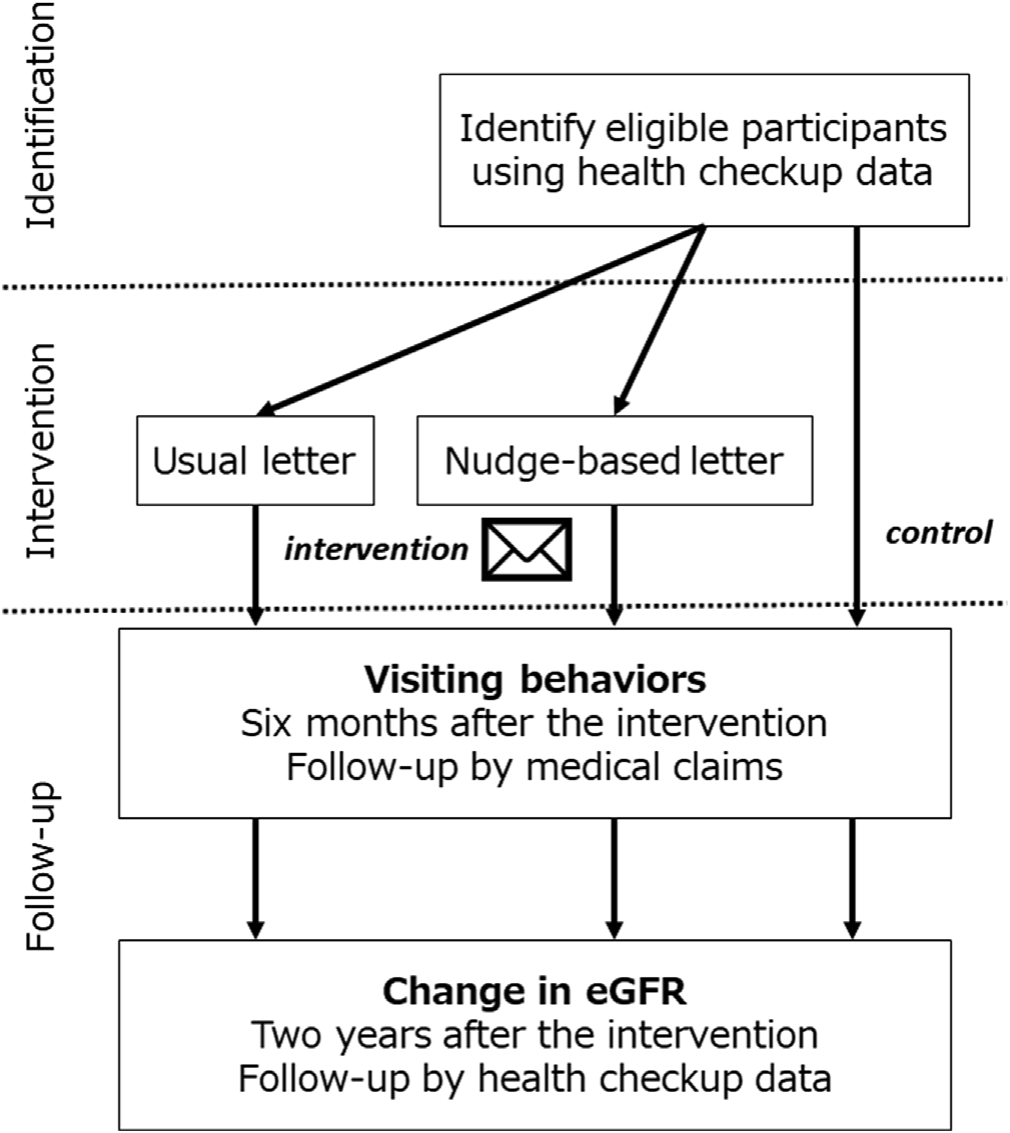
**Overall study design**. The pragmatic randomized controlled trial of nudging CKD patients after screening was designed in the health care system setting to examine the effect of a novel behavioral intervention among CKD patients. Identification phase: We will identify eligible CKD patients according to health checkup data. Intervention phase: We will randomize participants into three groups: “usual letter,” “nudge-based letter,” and control group. Follow-up phase: Between the groups, we will compare outcomes of patients' visiting behaviors to physicians and change in their kidney function according to medical claims and health checkup data. CKD, chronic kidney disease

The pragmatic randomized controlled trial (RCT) of nudging CKD patients after screening was designed in the health care system setting [11] to examine the effect of a novel behavioral intervention among CKD patients (Fig. 1). We will identify CKD patients at screening and randomize them into three groups: “usual letter,” “nudge-based letter,” and control group. Between the groups, we will compare outcomes of patients’ visiting behaviors to physicians (visiting physicians for CKD care for 6 months after the intervention) and change in their kidney function (change in the eGFR after 2 years).

### 2.2 Participants and setting

We conducted this pragmatic RCT among persons insured by the Health Insurance Association for Architecture and Civil Engineering companies in Japan (HIA^2^CE), which is a large health insurance association that insures about 400,000 persons as employees and their family members. The health insurance association covers most of the architecture and engineering companies all over Japan, from local small construction companies to major general contractors.

The inclusion criteria are (a) age between 40 and 63 years, (b) patients receiving CKD screening, and (c) those with an eGFR <60 mL/min/1.74 m^2^ or positive proteinuria. Proteinuria was measured by a urine dipstick test as −, ±, 1+, or 2+. Results of urine protein ≥1 + were defined as positive proteinuria. The exclusion criteria are (a) patients with an eGFR <15 mL/min/1.74 m^2^, and (b) those with ESRD. Those criteria will be confirmed using health checkup data from April 2018 to March 2019. The eGFR will be estimated from annual health checkup data using the Japanese coefficient-modified CKD Epidemiology Collaboration equation, which has been validated in the Japanese population [12,13].

### 2.3 Intervention and control

We defined two intervention groups that will receive a notification letter, the usual letter and nudge-based letter. We also defined a third group as the control group that will not receive a letter. Data collectors and those assessing outcomes will be blinded to the assignment of interventions.

In the usual letter, we provide information about CKD based on clinical evidence; recommend visits to physicians; and show patients' individual health checkup results of the eGFR, urine protein, and risk factors of hypertension (systolic blood pressure/diastolic blood pressure ≥140/90 mmHg or receiving antihypertensive drugs), diabetes (glycated hemoglobin A1c level ≥6.5% or receiving antidiabetic drugs), and smoking (current smoker or not). In the nudge-based letter, we encourage visits to physicians using the nudge approach in behavioral economics. The nudge is defined as “any aspect of the choice architecture that alters people's behavior in a predictable way without forbidding any options or significantly changing their economic incentives” [9]. In general, behavioral economics researchers consider people's seemingly irrational behaviors, procrastination [14] and loss avoidance [15], to construct a nudge-based intervention. In this study, to alter the participant's visiting behavior to physicians in a clinically desirable way without forbidding any other options, our nudge approach includes “commitment” (designing the space for intentions prompts, where participants write the visiting date and time, solidifying their intention of visiting physicians, and preventing the procrastination) [16] and “loss-framed message” (considering loss avoidance and giving information that emphasizes the loss of opportunity for preventing CKD progression and for work) [17]. We also add information about the concrete steps to visit a physician in order to decrease the participant's burden (cognitive costs). We also show individual health checkup results in the nudge-based letter as in the usual letter. The volumes of documents are similar between both the nudge-based letter and usual letter. Supplemental Fig. 1 and Supplemental Fig. 2 show the format of these letters. These letters will be sent by mail to individuals from the health insurance association. The research team is not involved in the individual shipping business of personal data, and the trial is open-labeled without blinding because it would be difficult to send a placebo letter to the control group.

Conventional follow-up after the screening will be implemented in all groups including the control group. In the Japanese health checkup system, all adults aged 40 years or older receive an annual health checkup with the aim of screening for metabolic syndrome. The participants receive notification of the abnormalities in the health checkup results regarding central obesity, hypertension, diabetes, and hyperlipidemia, but it does not include specific information about CKD. There is no special recommendation to visit physicians for CKD care in the conventional follow-up.

### 2.4 Randomization

For this pragmatic trial in the health care system, a research coordinator from the health insurance association assigned eligible participants and generated a unique number for each. After stratifying patients by the timing of screening (quarterly), eGFR (≥60 mL/min or <60 mL/min), and urine protein (≥+ or ≤±), we conduct random allocation with the permuted block method for each stratum. We randomize participants in a 2:2:1 ratio into the usual letter group, nudge-based letter group, and control group, respectively.

### 2.5 Outcomes

Our primary outcome is proportion of patients’ visiting physicians for 6 months after the intervention, and the secondary outcome is the change in the eGFR at 2 years after the intervention (Table 1). The primary outcomes of visiting physicians after the intervention is defined independently of the presence or absence of the visit before the intervention. Although our definition of visiting physicians after the intervention may include the follow-up visit (re-visit after the intervention with a history of visiting physicians before the intervention), this does not affect internal validity of the trial. Follow-up of participants will end at 2 years after their intervention data are collected or until they leave the HIA^2^CE.

**Table 1:** Outcomes.

Primary outcome
**Proportion of visiting** The proportion of participants who visit physicians for CKD care for 6 months after the intervention
**Secondary outcome**
**Change in the eGFR** Change in the eGFR (mL/min/1.73 m^2^) from the pre-intervention value to the post-intervention value after 2 years
**Proportion of continuous visiting** The proportion of participants who visit physicians for CKD care at least twice between 1 year and 2 years after the intervention

CKD, chronic kidney disease; eGFR, estimated glomerular filtration rate.

The medical claims data from the intervention date to 6 months after the intervention is used to define visiting behavior. If the participants have CKD-related diagnostic codes (Table 2) in their medical claims during this period, it is considered that they visited physicians for CKD care. The health checkup data of pre-intervention and post-intervention (2 years later) is used to define change in the eGFR.

**Table 2:** Chronic kidney disease-related diagnostic codes.

	ICD-10 codes
Chronic kidney disease	N170, N171, N172, N178, N179, N180, N188, N189, N19, N990
Tubulointerstitial nephritis	N110, N111, N118, N119, N12, N140, N141, N142, N143, N144, N150
Chronic glomerular nephritis	N002, N003, N004, N006, N007, N009, N012, N014, N016, N017, N019, N028, N029, N030, N032, N033, N034, N036, N037, N039, N040, N042, N044, N046, N049, N050, N051, N052, N053, N054, N055, N056, N057, N058, N059, N069, N079, N085
Diabetic nephropathy	E102, E112, E132, E142
Hypertensive nephrosclerosis	I129, I120
Polycystic kidney disease	Q613

### 2.6 Statistical analysis

#### 2.6.1 Sample size

Based on past medical claims data and results from the previous intervention, we assume that the proportions of patients visiting physicians in each group are 0.15, 0.20, and 0.25 in the control group, usual letter group, and nudge-based letter group, respectively. We set the allocation ratio as 1:2:2 for the control group, usual letter group, and nudge-based letter group, respectively. The sample sizes of 850, 1700, and 1700 patients for each group were determined to achieve ≥80% power overall and for all pairwise comparisons with the chi-square test by the simulation-based approach. The sample size provides ≥99% power to detect the difference of change from the baseline eGFR of −3 (standard deviation = 10) with a two-sided significance level of 0.05.

#### 2.6.2 Data analysis

We will describe participant characteristics with means and proportions by groups and compare them between groups using analysis of variance and chi-square tests, and their non-parametric analogs, as appropriate.

In our analysis to evaluate the effect of the intervention, we will conduct an intention-to-treat analysis. In our primary analysis, we will apply a logistic regression model with a dichotomous variable of visiting behavior as the dependent variable, and dummy variables of three groups and allocation adjustment factors (quarterly timing of the intervention, eGFR, and urine protein) as the independent variables. In the logistic regression model, we will estimate p-values by omnibus tests (Wald test) for the differences in outcome (proportion of visits to physicians) between the groups. If the two-sided p-values by omnibus tests is <5%, we will conclude that there are statistically significant differences in outcome between the groups and will continue to perform pairwise comparisons in order to adjust for multiple comparisons [18]. From the logistic regression model, we will estimate risk differences and their 95% confidence intervals using the model-based standardization technique [19]. In a sensitivity analysis, we will adjust for the presence of lifestyle guidance involved in the usual follow-up after the health checkup. We will also estimate the proportion and its Wald-type 95% confidence interval of the primary outcome for each group. Further, we will estimate the odds ratio and its 95% confidence intervals, and compare them between the groups.

For the confirmatory analysis of the secondary outcome, we will apply the closed testing procedure. Specifically, only if the two-sided p-values by the omnibus test of the primary outcome are <5%, we will conduct the following analysis on the difference of the eGFR. We will apply a linear regression model with the difference of the eGFR as the dependent variable, and dummy variables of the three groups and allocation adjustment factors (quarterly timing of the intervention, eGFR, and urine protein) as the independent variables. We will estimate p-values by omnibus tests (Wald test) to determine differences in outcome (i.e., the difference of eGFR) between the groups. If the two-sided p-values by omnibus tests are <5%, we will conclude that there are statistically significant differences in outcome between the groups and will continue to conduct pairwise comparisons.

In the subgroup analysis, we will assess the effect of the interventions by the presence of diabetes, age categories (40–49, 50–59, and ≥60 years), sex, and past CKD detection.

### 2.7 Ethical statements

We are conducting the pragmatic RCT in cooperation with conventional health promotion activities by the health insurance association. In this study, we are using routinely collected data, not collecting additional data, to identify eligible participants, follow them, and assess their outcomes. The intervention just involves receiving letters to encourage patients to visit physicians without any obligation. Therefore, we received approval from the institutional review board of Kyoto University (approval number: C1420), and the need for informed consent from the participants was waived.

## 3 Results

**Fig. 2:**
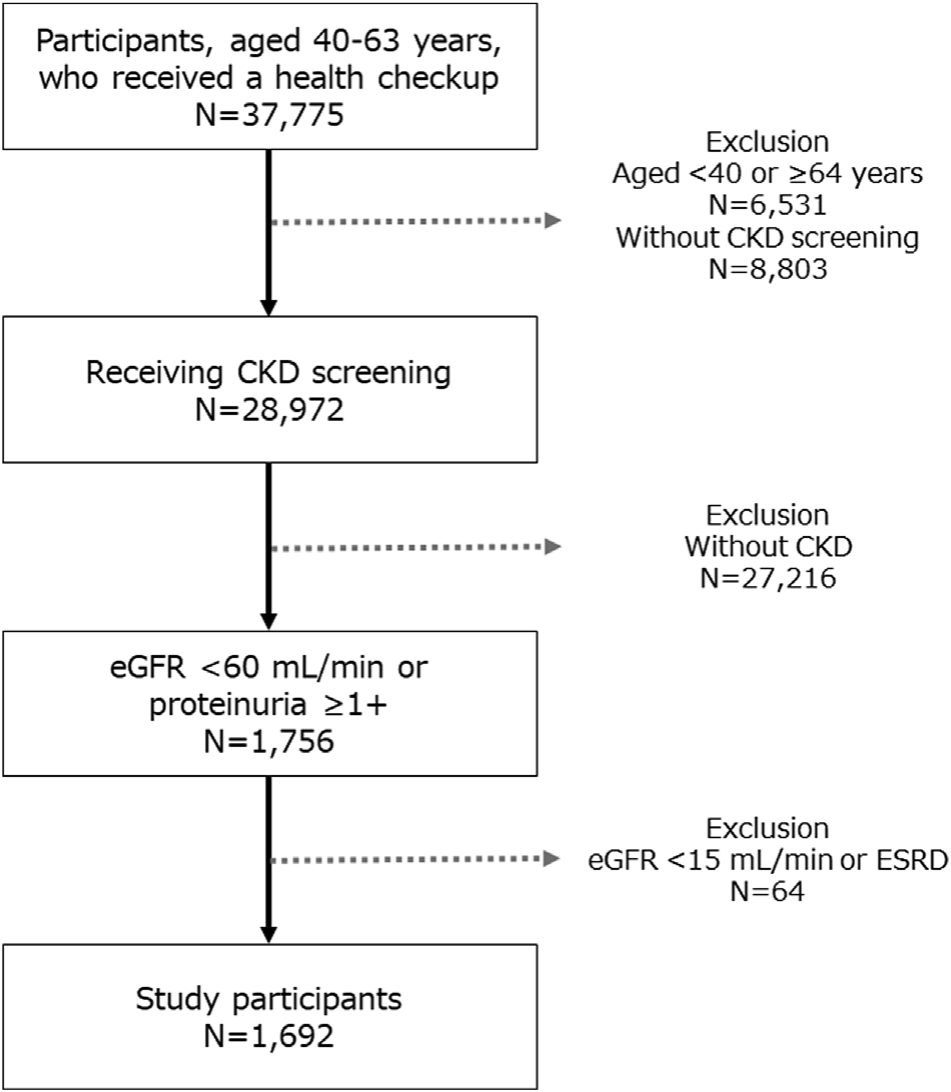
**Selection process of study participants, from 2018 April to 2018 June.** Among 37,775 participants, aged between 40 and 63 years, who received a health checkup between April and June in 2018, we have enrolled 1,692 CKD participants (4.5%). CKD, chronic kidney disease; eGFR, estimated glomerular filtration rate; ESRD, end stage renal disease.

Among 37,775 participants, aged between 40 and 63 years, who received a health checkup between April and June in 2018, we have enrolled 1,692 CKD participants (4.5%). The selection process of study participants during this period is summarized in Fig. 2. Then we randomized 677 participants into the usual letter group, 677 participants into the nudge-based letter group, and 338 participants into the control group. We confirmed that a large number of eligible participants for the trial could be identified by routinely collected health checkup data. Table 3 shows participant characteristics in each group. Middle-aged men are the focus of our study participants. Age, eGFR, proportion of patients with urine protein ≥1+, blood pressure, and HbA1c level were similar between the groups. The proportion of patients with a previous visit was slightly higher in the control group than in the intervention group (no statistical test). We will continue to select eligible participants by the end of March 2019 and follow them until their health check-up at 2 years later.

**Table 3:** Participant characteristics.

	Control group (N = 338)	Usual letter group (N = 677)	Nudge-based letter group (N = 677)
Age, mean (SD), years	53.7 (6.6)	53.8 (6.5)	53.6 (6.6)
Male sex, n (%)	313 (92.6)	617 (91.1)	623 (92.0)
eGFR, mean (SD), mL/min/1.73 m^2^	68.7 (15.2)	68.1 (16.0)	68.7 (15.9)
Urine protein ≥+, n (%)	214 (63.3)	429 (63.4)	429 (63.4)
SBP, mean (SD), mmHg	129.8 (17.9)	129.9 (18.7)	130.2 (18.7)
DBP, mean (SD), mmHg	81.9 (13.0)	82.2 (12.9)	82.0 (13.0)
HbA1c, mean (SD), %	6.1 (1.1)	6.0 (1.1)	6.10 (1.22)
Hypertension, n (%)	138 (37.2)	252 (40.8)	264 (39.0)
Diabetes, n (%)	66 (19.5)	100 (14.8)	108 (16.0)
Current smoking, n (%)	120 (35.5)	217 (32.1)	229 (33.8)
Previous visiting,^a^ n (%)	19 (5.6)	31 (4.6)	28 (4.1)

SD, standard deviation; eGFR, estimated glomerular filtration rate; SBP, systolic blood pressure.

DBP, diastolic blood pressure; HbA1c, glycated hemoglobin A1c; CKD, chronic kidney disease.

a Previous visiting was defined as proportion of visiting to physicians within 6 months before health checkup.

## 4 Discussion

Large-scale CKD screening is criticized from the viewpoint of cost-effectiveness [20], but consensus has been obtained that the prevention of CKD progression is an important public health issue. Because Japan is a country where pre-diagnosis CKD data have been accumulated by nationwide health checkups, we could develop an efficient intervention after CKD screening and assess its effectiveness in the real world. Conventional CKD screening in Japan can detect potential CKD patients from the general population but lacks intervention to make them visit physicians after the screening. Consequently, we designed a pragmatic trial of nudging CKD patients to visit physicians after screening. The results from this trial will contribute to filling the gap between screening and subsequent medical interventions and will also be a model for a “learning health system” that utilize the routinely collected health data to improve the health care system.

Our pragmatic RCT is designed in the health care system. By taking advantage of having large-scale health checkup data in Japan, we are conducting this implementation research to improve patients' visiting behaviors to physicians after CKD screening. Since it is usually difficult to allocate intervention, i.e., whether to provide medical treatment, in a high-risk population, our intervention to encourage patients to visit physicians by just sending them a letter is a feasible and realistic approach for the prevention of CKD progression. Our intervention is inexpensive and can be implemented for other health issues in the future. We will only use routinely collected data from the health care system to identify eligible participants, follow them, and measure their outcomes. This makes it possible to perform a large-scale pragmatic RCT involving a wider population at a lower cost. Our pragmatic RCT can assess both short-term outcomes of patients’ visiting behavior and long-term outcome of renal function through analysis plans, in which we can manage statistical multiplicity.

Another major feature of this trial is to examine the effect of a nudge approach on patients' behaviors in one important issue of public health. Many of the interventional studies in public health have focused on the effect of mandatory interventions (restricting a patient's choice) or financial incentives. Additionally, these studies have been conducted on some limited issues, including smoking and obesity. In behavioral economics, an increasing number of studies has investigated the effect of the nudge approach, especially in the United States and Europe [16,[21], [22], [23]]. However, many of these studies have also focused on smoking and obesity, or when studying the other clinical issues, the study subjects have usually been limited to patients in a certain hospital. Therefore, the present trial is unique in that it is investigating the effect of the nudge approach in Japan, an unexamined clinical issue, and evaluating subjects from a wider range of regions.

Investigating the effect of the nudge approach is recommended by the Japanese government, which followed the Behavioral Insights Team in the United Kingdom and founded the Behavioral Sciences Team in 2017, to apply behavioral economics and nudge in various policy fields, including health policy. As we explained earlier, we considered patients’ behavioral characteristics and designed the letter accordingly. Thus, our nudge approach can be expected to be cost-effective.

There are several limitations to this pragmatic RCT. First, we will use only routinely collected data. Avoiding additional data collection dramatically decreases the cost and effort, and enables such a large-scale pragmatic RCT to be conducted. However, we cannot assess unmeasured factors, which are not included in the health checkup and medical claims. Second, we will use a one-time measurement of kidney function to identify CKD participants at screening. The original definition of CKD is a continuous renal function deficiency, which needs at least two time measurements of kidney function [24,25]. In previous clinical studies, a one-time measurement of kidney function is often used to define CKD, which is validated as a risk factor in identifying the population with renal risk [26,27]. Further, our intervention to encourage potential CKD participants to visit physicians is clinically reasonable to confirm the presence of CKD by performing re-tests of kidney function. Third, we will define our outcomes of visiting behavior by CKD-related diagnostic codes in medical claims data, and thus, patients who receive CKD care without CKD-related diagnostic codes will be misclassified. For example, CKD patients with hypertension might receive CKD care without CKD-related diagnostic codes. However, we expect this misclassification to be a non-difference between allocation groups, and the effect of the intervention will not be biased by this factor. Fourth, the trial is open-labeled without blinding. However, because we will use routinely collected data to measure study outcomes based on a pre-specified definition, outcome assessment will be less susceptible to non-blinding. Fifth, some participants are excluded because of the absence of CKD screening. In Fig. 2 and 92.3% of participants received CKD screening. Whether a participant receives CKD screening in addition to the mandatory health checkup items is decided by each company and cannot be decided by the individual participant. Therefore, the effect of excluding a small portion of participants without CKD screening is limited. Finally, we will include the middle-aged male-focused Japanese population in this trial. Although this pragmatic RCT in the health care system will include a broader population than a conventional trial, generalizability to other populations is limited.

## 5 Conclusions

With prevalence of 4.5% for CKD and only 7–8% for visiting physicians before screening, it is apparent that the patients who are not under the care of a physician continue to have deteriorating kidney function. We anticipate that with an increase in the proportion of patients visiting physicians following screening, clinical management will be instituted in time and patient outcomes optimized. This will also ensure returns on investment in CKD screening programs. The intervention we will examine is a low-cost method that can be implemented to other health issues in the future. The trial will also show the model for utilizing routinely collected data to assess the effect of a public health intervention in the real world.

## Funding

This work is supported by the Japan Society for the Promotion of Science KAKENHI (grant number: 16K19251). The funding sources had no involvement in conducting the study.

## Declaration of conflicting interests

The Authors declare that there is no conflict of interest.

## Trial registration number

UMIN-CTR R000039631.
