# Supplementary material for: Nudging patients with chronic kidney disease at screening to visit physicians: A protocol of a pragmatic randomized controlled trial
Source: Contemp Clin Trials Commun. 2019 Aug 16;16:100429. doi: 10.1016/j.conctc.2019.100429 (PMC6722278; doi:10.1016/j.conctc.2019.100429)
Supplement: Multimedia component 1 [file mmc1.pdf]

Supplemental Figure 1. Design of letters for the intervention

A) Usual letter (Japanese)

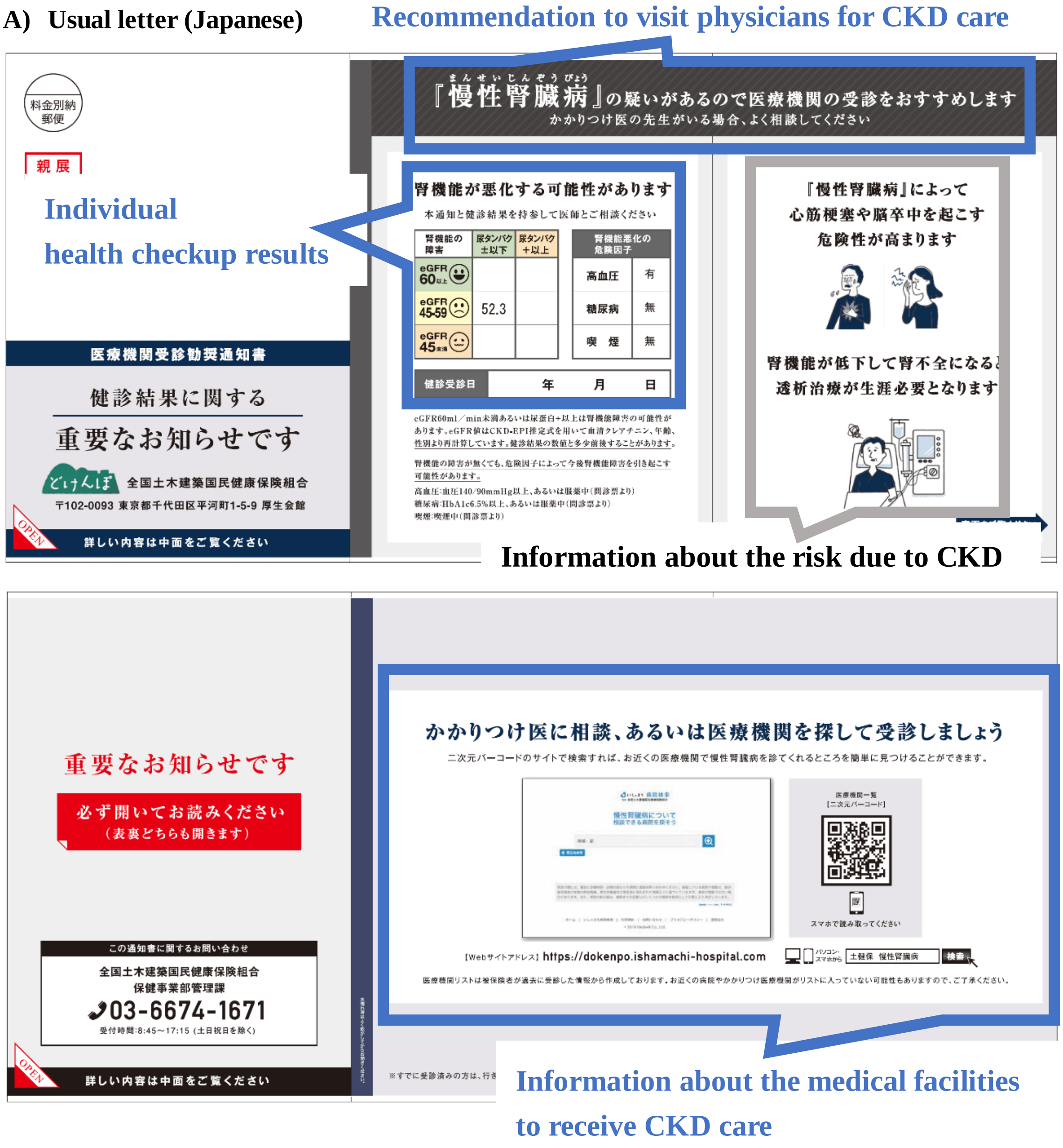

In the usual letter, we provide information about CKD based on clinical evidence; encourage visits to physicians; and show patients' individual health checkup results of the eGFR, urine protein, and risk factors of hypertension (systolic blood pressure/diastolic blood pressure ≥140/90 mmHg or receiving antihypertensive drugs), diabetes (glycated hemoglobin A1c level ≥6.5% or receiving antidiabetic drugs), and smoking (current smoker or not). eGFR, estimated glomerular filtration rate; CKD, chronic kidney disease

B) Nudge-based letter (Japanese)

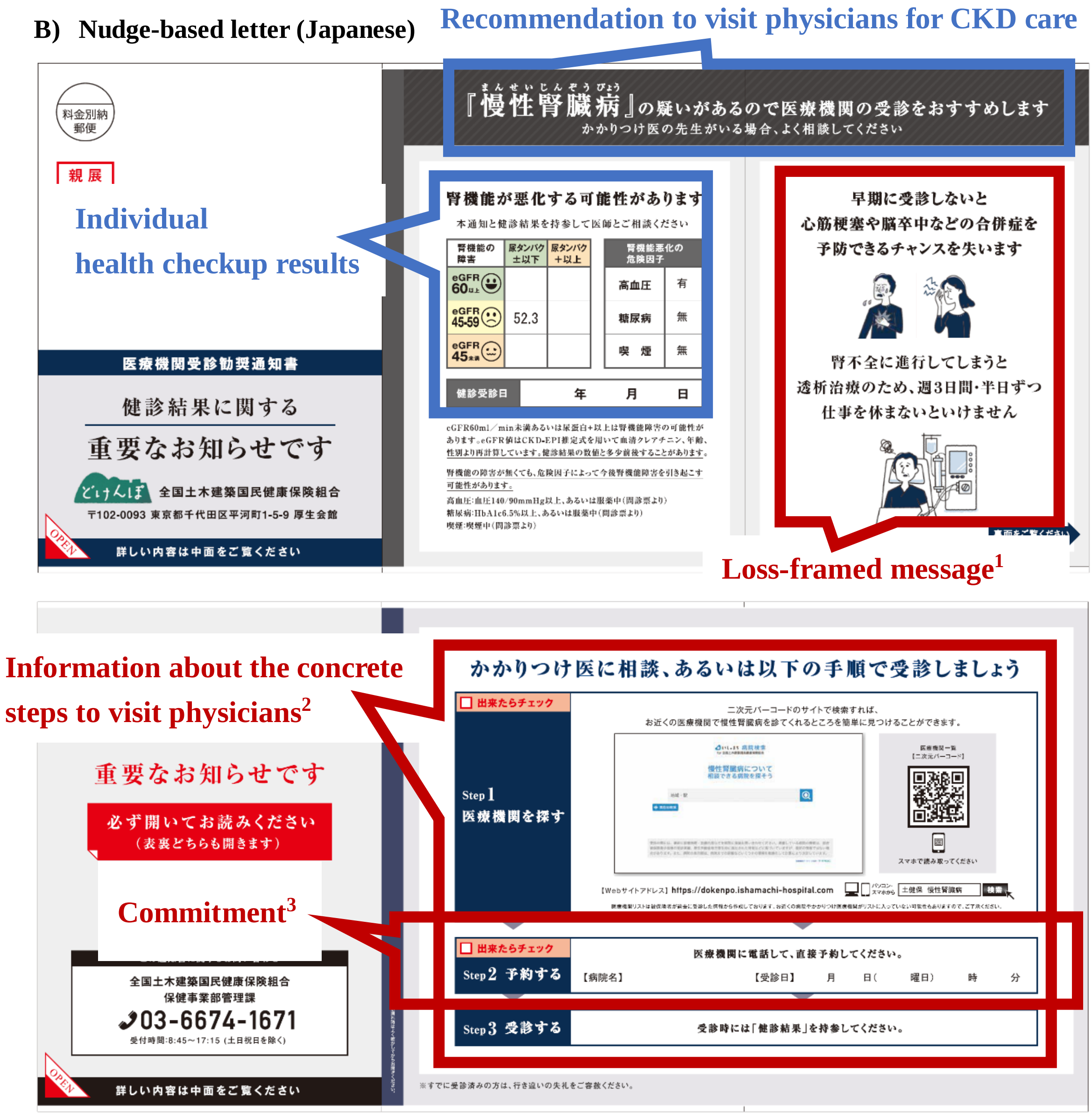

In the nudge-based letter, we encourage visits to physicians using the following nudge approaches.

1. Loss-framed message

   If you don’t see a doctor early, you will lose the chance to prevent complications such as myocardial infarction and stroke. If kidney failure progresses, you have to take a half-day off work 3 days a week for dialysis treatment.

2. Information about the concrete steps to visit physicians

   Step 1: Please search medical institution at the website.

   Step 2: Please call the medical institution and make a reservation directly.

   Step 3: Please visit the medial institution.

3. Commitment: Please declare hospital name, and visiting date and time.

Supplemental Figure 2. An example of contents in a usual follow-up letter after the screening

Report of screening results			
		Results	Comments
Physical measurement	Height	165.8 cm	
	Weight	77.4 kg	Mild obesity
	Standard weight	60.4 kg	
	BMI	28.2 kg/m^2	Mild obesity
	Waist circumference	88.9 cm	High
Urine test	Urine protein level	–	
	Urine sugar level	–	
Blood pressure		144/92 mmHg undertreatment	High
Blood lipid test	LDL cholesterol level	128 mg/dL	
	Triglyceride level	69 mg/dL	
Renal function	Serum creatinine level	1.20 mg/dL	High
	eGFR	50.3 mL/min	Low
Uric acid	Uric acid level	4.6 mg/dL	
Blood glucose	HbA1c level	5.5%	
Blood test	White blood cell count	7600/μL	
	Hemoglobin level	14.7 g/dL	

BMI, body mass index; LDL, low-density lipoprotein; eGFR, estimated glomerular filtration rate; HbA1c, glycated hemoglobin

Summary comments

You have mild obesity.<br>Please be careful about your lifestyle.<br>Please pay attention to abnormal results and comments.<br>Please follow your doctor's instructions if necessary.
